# Characterization of Ectonucleotidases in Human Medulloblastoma Cell Lines: ecto-5′NT/CD73 in Metastasis as Potential Prognostic Factor

**DOI:** 10.1371/journal.pone.0047468

**Published:** 2012-10-18

**Authors:** Angélica Regina Cappellari, Liliana Rockenbach, Fabrícia Dietrich, Vanessa Clarimundo, Talita Glaser, Elizandra Braganhol, Ana Lúcia Abujamra, Rafael Roesler, Henning Ulrich, Ana Maria liveira Battastini

**Affiliations:** 1 Departamento de Bioquímica, Instituto de Ciências Básicas e da Saúde, UFRGS, Porto Alegre, Rio Grande do Sol, Brasil; 2 Departamento de Bioquímica; Instituto de Química, São Paulo, São Paulo, Brasil; 3 Centro de Ciências Químicas, Farmacêuticas e de Alimentos, UFPel, Pelotas, Rio Grande do Sol Brasil; 4 Laboratório de Pesquisa em Câncer; Hospital de Clínicas de Porto Alegre, UFRGS, Porto Alegre, Rio Grande do Sol, Brasil; 5 Instituto do Câncer Infantil do RS, ICI-RS, Porto Alegre, Rio Grande do Sol, Brasil; 6 Departamento de Farmacologia, Instituto de Ciências Básicas da Saúde, UFRGS, Porto Alegre, Rio Grande do Sol, Brasil; 7 Instituto Nacional de Ciência e Tecnologia Translacional em Medicina, UFRGS, Porto Alegre, Rio Grande do Sol, Brasil; University of Colorado Denver, United States of America

## Abstract

Medulloblastoma (MB) is the most common malignant brain tumor in children and occurs mainly in the cerebellum. Important intracellular signaling molecules, such those present in the Sonic Hedgehog and Wnt pathways, are involved in its development and can also be employed to determine tumor grade and prognosis. Ectonucleotidases, particularly ecto-5′NT/CD73, are important enzymes in the malignant process of different tumor types regulating extracellular ATP and adenosine levels. Here, we investigated the activity of ectonucleotidases in three malignant human cell lines: Daoy and ONS76, being representative of primary MB, and the D283 cell line, derived from a metastatic MB. All cell lines secreted ATP into the extracellular medium while hydrolyze poorly this nucleotide, which is in agreement with the low expression and activity of pyrophosphate/phosphodiesterase, NTPDases and alkaline phosphatase. The analysis of AMP hydrolysis showed that Daoy and ONS76 completely hydrolyzed AMP, with parallel adenosine production (Daoy) and inosine accumulation (ONS76). On the other hand, D283 cell line did not hydrolyze AMP. Moreover, primary MB tumor cells, Daoy and ONS76 express the ecto-5′NT/CD73 while D283 representative of a metastatic tumor, revealed poor expression of this enzyme, while the ecto-adenosine deaminase showed higher expression in D283 compared to Daoy and ONS76 cells. Nuclear beta-catenin has been suggested as a marker for MB prognosis. Further it can promotes expression of ecto-5′NT/CD73 and suppression of adenosine deaminase. It was observed that Daoy and ONS76 showed greater nuclear beta-catenin immunoreactivity than D283, which presented mainly cytoplasmic immunoreactivity. In summary, the absence of ecto-5′NT/CD73 in the D283 cell line, a metastatic MB phenotype, suggests that high expression levels of this ectonucleotidase could be correlated with a poor prognosis in patients with MB.

## Introduction

Medulloblastoma (MB) is the most common malignant brain tumor in children. It affects preferentially boys around 9 years of age, with few cases reporting MB among adults of over 20 years of age. MB occurs mainly in the cerebellum and posterior fossa, being able to migrate into other brain structures. This tumor generates extraneural metastasis in 7.1% of the cases, which are related to its poor prognosis [1], [2]. The World Health Organization (WHO) has classified MB as a grade IV tumor, the most malignant grade [3]. This tumor is subdivided into subsets including desmoplastic/nodular, anaplastic, MB with extensive nodularity, and large cell MB [3], [4]. Sonic Hedgehog, C-MYC, NOTCH and Wnt induce the main intracellular signaling pathways involved in MB development, and are related to the different MB subsets and tumor grade [4]–[6]. Mutations in downstream effectors of the Wnt pathway, including the APC protein and β-catenin, presented in sporadic MB suggest that an aberrant pathway signaling participates in disease development. Further, recent studies showed that nuclear or cytoplasmic β-catenin immunoreactivity could be related with a good or bad prognosis of patients with MB, respectively [7].

Numerous factors are known to influence malignant tumor progression. Among them, we can highlight the purinergic system, which has been identified in many tumor types [8]–[11]. ATP is a nucleotide which can be released into the extracellular medium in response to physiological or pathological conditions and perform different functions by activating P2X, determined as ionotropic or ligand-gated receptor class, and P2Y receptors, classified as metabotropic G-protein coupled receptors [12], [13].

ATP signaling is modulated by a general class of efficient enzymes denominated ectonucleotidases. They are subdivided into E-NTPDases (ecto-nucleotide triphosphate diphosphohydrolases), E-NPP (ecto-nucleotide pyrophosphate/phosphodiesterase), Ecto-5′NT/CD73 (Ecto-5′nucleotidase/CD73) and ALP (ecto-alkaline phophatase) [14]. NTPDases1, 2, 3 and 8 are ectoenzymes, which differ in their substrate preference between ATP and ADP, as well as in their tissue distribution. Intracellularly expressed NTPDase5 and 6 are secreted into the extracellular medium upon stimulation, while NTPDases4 and 7 are exclusively intracellular enzymes localized in the lumen of organelles. The E-NPP family is constituted by seven ectoenzymes, but only NPP1, 2 and 3 are involved in purinergic signaling due to their capacity to hydrolyze a wide spectrum of phosphate substrates, such as conversion of ATP into AMP and PPi and ADP into AMP and Pi [14]–[16].

The Ecto-5′NT/CD73 is a widely distributed enzyme, bound to the plasma membrane by a glycosyl-phosphatidylinositol lipid anchor. Its main function is to hydrolyze AMP into adenosine in the extracellular medium. Additionally, this enzyme exerts non-enzymatic functions by participating in cell-cell and cell-matrix interactions and stimulating intracellular signaling pathways [15], [17], [18]. Ecto-alkaline phophatases (ALPs) are composed by four enzymes broadly distributed in the human body. They hydrolyze a wide substrate variety included ATP, ADP, AMP and PPi [19]. However, ALPs comprise an important group of enzymes that mainly hydrolyze AMP, participating in the extracellular metabolism of nucleotides as an adenosine producer [20], [21]. Finally, extracellular adenosine availability is controlled by plasma-membrane-located adenosine transporters and/or by the action of ecto-adenosine deaminase (ADA) producing inosine [22].

In the last years, many reports have shown the involvement of the purinergic system in tumor progression, with focus on the participation of ectonucleotidases in the pathogenesis of this process. Our previous findings show that cell lines with high malignant grade, present elevated ecto-5′NT/CD73 and no E-NTPDase activity [23]. The absence of E-NTPDases results in accumulation of ATP [24] and the presence of ecto-5′NT/CD73 favors adenosine production from AMP which is liberated by the death of normal tissue surrounding the tumor [25]. Both, ATP and adenosine, *in vitro* stimulated glioma cell proliferation [26]. Additionally, gliomas present a positive correlation between an increase in cell confluence with increased ecto-5′NT/CD73 expression, suggesting the participation of this enzyme in progression of this tumor [9].

The MB cell lines used in this study are considered important models for evaluating progression and malignancy [27]–[30]. The Daoy and ONS76 cell lines are considered to be representative of a primary MB. On the other hand, the D283 cell line is representative of a MB that formed metastases in the peritoneum, from which this cell line was derived. In view of that, we proposed characterize the profile of ectonucleotidases expression and activity in MB cell lines. Here we report that Daoy, ONS76 and D283 cell lines differ in levels of ectonucleotidase expression and enzymatic activity. We also showed that variation in the ecto-5′NT/CD73 and ADA expression can be associated with correspondent β-catenin immunoreactivity levels, suggesting the importance of these enzymes as prognosis markers.

## Materials and Methods

### Reagents

Cell culture medium, penicillin/streptomycin, trypsin/EDTA solution and fetal bovine serum were purchased from Gibco (Gibco BRL, Carlsbad, CA, USA). ATP, ADP and all monophosphate nucleosides, adenosine, thymidine 5′-monophosphate ρ-nitrophenyl ester sodium salt, 4-Nitrophenol, *β*-glycero-phosphate, *D-*glucose 6-phosphate sodium salt and levamisole were obtained from Sigma (Sigma Aldrich Corp., St. Louis, USA). All other chemicals utilized were of analytical grade.

### Cell Culture

Daoy and ONS76 cell lines (representative of a human primary tumor) and D283 (representative of a human metastatic MB) were kindly donated by Cancer Research Laboratory and Childreńs Cancer Institute (HCPA and ICI-RS, Rio Grande do Sul, Brazil). The Daoy and D283 cell lines were originally obtained from the ATCC (American Type Culture Collection) while the ONS76 cell line was originally from JCRB (Japanese Collection of Research Bioresources). The cells were grown and maintained in Dulbeccós Modified Eaglés Medium (DMEM) low glucose, containing 0.5 U/mL penicillin/streptomycin antibiotics and supplemented with 10% fetal bovine serum (FBS). Cells were kept at 37°C, in an incubator with minimum relative humidity of 95% and 5% of CO_2_ in air.

### Nucleotide and Nucleoside Analysis by HPLC

Cells were seeded at density of 10^4^ cells/well in 24-multiwell plates. After reaching confluence, the cells were washed three times with incubation medium containing 2 mM MgCl_2_, 120 mM NaCl, 5 mM KCl, 10 mM glucose, 20 mM Hepes, pH 7.4. The reaction was started by adding AMP at a final concentration of 100 µM in 250 µL at 37°C. After different incubation times (0, 5, 10, 20, 30, 60, 90 min) the supernatant was taken and maintained on ice. For determination of basal nucleotide secretion, cells were incubated for 90 min with incubation medium, without substrate. The collected supernatants were centrifuged for 30 min at 16.000×g and 4°C, and aliquots of 40 µL were analyzed with a reversed-phase HPLC system (Supelcosil LC-18, 25 cm×4.6 mm, Supelco) in a Shimadzu Instruments Liquid Chromatograph (Shimadzu, Japan). Separation was performed by using a linear gradient from 100% of solvent A (60 mM KH_2_PO_4_ and 5 mM tetrabutylammonium phosphate, pH 6.0) to 100% of solvent B (70% 100 mM KH_2_PO_4_ and 5 mM tetrabutylammonium phosphate, pH 6.0 in 30% methanol). The quantities of purines were detected by absorption at 254 nm and all peaks were identified by its retention time and quantified by comparison with standards. Purine concentrations are expressed as nmol product/mg protein (mean ± SD). All assays were carried out in triplicates, and the controls utilized to correct the non-enzymatic hydrolysis of nucleotides were done by measuring the peaks present in the same reaction medium in the absence of cells.

### Reverse Transcriptase-PCR and Real-time PCR

Total RNA from MB cell line cultures was isolated with Trizol LS reagent in accordance with the manufacturer’s instructions. The RNA samples were treated with DNAse (Deoxyribonuclease I Kit amplification – Sigma-Aldrich Brasil Ltda) for 10 min at 37°C and samples were incubated for 10 min at 65°C to stop the reaction. The cDNA was synthesized with RevertAid reverse transcriptase (Fermentas – Life Sciences) from 3 µg of total RNA in a final volume of 20 µL with an oligo dT primer in accordance with the manufacturer’s instructions. In the sequence, cDNA reactions were carried out for 1 hour at 42°C and stopped by heating to 70°C. Reaction mixes were used for RT-PCR in a total volume of 25 µL which included 1 µL of the RT reaction, 2.5 µL 10× Taq DNA Polimerase Buffer, 1.5 µL of 25 mM MgCl_2_, 0.5 µL of 10 µM dNTP mix, 1 µL of each specific primer at 10 µM (Table 1) and 1 µL of Taq DNA polymerase enzyme (Fermentas – Life Sciences). The PCR cycling conditions were as follows: 2 minutes at 95°C, 30 seconds at 95°C, 30 seconds at the annealing temperature and 1 minute at 72°C. All PCR reactions were carried out for 35 cycles and included a final 10-minute extension at 72°C. Ten microliters of the PCR reaction were analyzed on a 2.0% agarose gel containing ethidium bromide and visualized under ultraviolet light. As a control for cDNA synthesis, glyceraldehyde 3-phosphate dehydrogenase (GAPDH) PCR was performed. Plasmids containing the sequences for NTPDase1, NTPDase2, NTPDase3 and ecto-5′-nucleotidase/CD73 were used as positive expression controls for these enzymes; T24 cDNA was used as a positive control for NTPDase5 gene expression. For analysis of NPP1, 2 and 3, ALP and ADA gene expression, we used a mix of human cell lines cDNA. Negative controls were performed by substituting the templates for DNAse/RNAse-free distilled water in each PCR reaction.

**Table 1:** Ectonucleotidases primer sequences for PCR experiments.

	*Primer sequence*	*(T°C)*	*Fragment size (bp)*
NPP1 F	5′- TGG GTT GAA ACC AAG CTG TGC CA -3′	69	94
NPP1 R	5′- ACA GGC AGC ATC ACA GCG ACA -3′		
NPP2 F	5′- AGG AGG AGC TCG TTC CAG T -3′	59	116
NPP2 R	5′- TCC CAT CCT TCT GCT CTC TT -3′		
NPP3 F	5′- GCA GGT GGA CCA GTC AGT GCC -3′	69	90
NPP3 R	5′- CCG CTG CTT CAG GCC TTC CA -3′		
NTPDase1 F	5′- AGT ATG GGA TTG TGC TGG ATG -3′	54	147
NTPDase1 R	5′- TCT GAA CAA ATT TTG AGA TTC CAG -3′		
NTPDase2 F	5′- CAG GAT GTG CCC AAA GAG A -3′	61	685
NTPDase2 R	5′- CCC CAT TGA AAG AGC ATC G -3′		
NTPDase3 F	5′- TTT CCC TGG ACA CCT TCA AC -3′	55	184
NTPDase3 R	5′- TGT ATT TGG GGC CAA GTC TC -3′		
NTPDase5 F	5′- GCA TTT GCC AAC ACC TTT TT -3′	55	179
NTPDase5 R	5′- ACA GGG CTC TCT GTG ATG CT -3′		
Ecto-5′NT/CD73 F	5′- AGG GGT GTG GAC GTC GTG GT -3′	65	84
Ecto-5′NT/CD73 R	5′- CCC AGC AGG CAC CTC TTT GGA -3′		
ADA F	5′- GGG GAG CGA GAC TTC CGG GGT -3′	69	83
ADA R	5′- TCC ACC ACC TTG GGG GAC CA -3′		
ALP F	5′- CAT TGG CAC CTG CCT TAC TA -3′	59	104
ALP R	5′- GCT CCA GGG CAT ATT TCA GT -3′		
GAPDH F	5′- TTC TTT TGC GTC GCC AGC CG -3′	64	93
GAPDH R	5′- ACC AGG CGC CCA ATA CGA CC -3′		

Primer sequences for PCR experiments. These primers listed here were used for both RT-PCR and real time PCR reactions with exception of primers for amplification of NTPDase2 coding sequences. Melting curve analysis was performed to determine the specificity for each real-time PCR reaction.

Real time PCR analysis was performed in the ABI Step One Plus Instrument (Applied Biosystems, Foster City, CA) using the Sybr Green amplification System. Each reaction was performed in a final volume of 25 µL containing 1 µL of the cDNA reversed transcribed from 3 µg RNA, 12.5 µL of the Sybr Green Universal PCR Master Mix (Applied Biosystems, Foster City, CA), and 0.3 µL of each forward and reverse primers (10 µM) (Table 1). The real time PCR reactions were performed using the following temperature protocols: 95°C for 10 minutes, 40 cycles of 95°C for 15 seconds each and 60°C for 1 minute. As the efficiency of all reactions was >95%, the ΔΔCt parameter was used for determination of relative expression data, taking GAPDH gene expression as an endogenous control for normalization. Standard curves were measured for each primer set and cDNA sample in order to verify reaction efficiency.

### Assay for Ectonucleotide Pyrophosphatase/phosphodiesterase Activity

E-NPP activity was assessed using 0.5 mM thymidine 5′-monophosphate ρ-nitrophenyl (5′-TMP-p-Nph), a substrate marker that is routinely used for the *in vitro* assay of these enzymes, as previously described [31]. The cells were incubated in a reaction medium containing 50 mM Tris-HCl buffer (pH 7.4 or 8.9), 5 mM KCl, 120 mM NaCl, 0.5 mM CaCl_2_, 60 mM glucose and 0.5 mM of 5′-TMP-p-Nph. The reaction was stopped with 200 µL of 0.2 M NaOH. The amount of released ρ-nitrophenol was determined as changes in optical density (OD) at 400 nm in a microplate reader. Specific activity was expressed as nmol ρ-nitrophenol/mg of protein.

### Ectonucleotidase Enzymatic Assay

For determination of nucleotide hydrolysis, 24-multiwell plates containing Daoy, ONS76 and D283 MB tumor cells were washed three times with phosphate-free incubation medium in the absence of the nucleotides. The reaction was started by addition of 200 µL of incubation medium containing 2 mM CaCl_2_, 120 mM NaCl, 5 mM KCl, 10 mM glucose, 20 mM HEPES (pH 7.4) and 1 mM of ATP or ADP at 37°C. Experimental conditions were the same as described above for quantifying monophosphonucleoside and phosphate ester hydrolysis, except that 2 mM MgCl_2_ was used instead of CaCl_2_. The substrates tested included AMP, CMP, GMP, IMP, UMP, inorganic pyrophosphate (PPi), glucose-6-phosphate (Glu-6P) and β-glycerophosphate (β-Gly). Substrate concentrations and the incubation times were chosen to assure the linearity of the reaction. The reaction was stopped by withdrawing an aliquot of the incubation medium and transferring it to a pre-chilled tube containing TCA (10% w/v). The inorganic phosphate (Pi) released was measured using the malachite green method [32], using KH_2_PO_4_ as a Pi standard. Controls to determinate non-enzymatic Pi released were performed by incubating cells in the absence of the substrate in absence of the cells. All samples were performed in triplicates. Specific activity was expressed as nmol Pi released/min/mg of protein (nmol Pi/min/mg).

### Protein Determination

For quantification of protein concentration, cells were dried, solubilized with 100 µL of 1 N NaOH and frozen overnight. The protein concentration was measured by the Coomassie blue method [33] using bovine serum albumin (BSA) as standard.

### Flow Cytometry Analysis

For flow cytometry analysis, Daoy, ONS76 and D283 MB cancer cell lines were maintained in culture flasks until they reach confluence. Cells then were trypsinized, dissociated and counted immediately in a hemocytometer. Then 1×10^6^ cells were centrifuged for 5 min at 400 g and washed twice with phosphate buffered saline (PBS) plus 1% fetal calf serum (FCS). The sediments were suspended and incubated for 1 h with purified mouse anti-human CD73 antibody (BD Pharmingen TM) (1∶10) for 1 h at 4°C. In parallel, the same number of cells was incubated without antibodies. In sequence, all samples were washed with PBS plus 1% FCS and centrifuged. The pellets were suspended and incubated for 1 h with Alexa fluor 555 rabbit anti-mouse IgG (1∶100). After this, labeled cells were washed with PBS and immediately analyzed by flow cytometry (FACS Calibur, BD Biosciences, Mountain View, CA). Ten thousands events in the cell gate were collected and further analyzed using the CellQuest software (BD Biosciences) and FCS Express 4 software.

### Extraction of Nuclear and Cytosolic Fractions and Western Blotting Assays

For preparation of cell lysates, MB cells were trypsinized, centrifuged for 10 min at 400 g, washed with PBS and centrifuged again. The pellet was then dissolved in lysis buffer (10 mM Hepes, 1.5 mM MgCl_2_, 10 mM KCl, 0.1 mM EDTA, 0.1 mM EGTA, 0.5% NP40) plus a protease inhibitor cocktail (Fermentas – Life Sciences) and phosphatase inhibitors (2 mM orthovanadate and 5 mM sodium fluoride), incubated for 15 min on ice, and then centrifuged for 5 min at 13,000 g and 4°C. The supernatant was collected and used as the soluble cytosolic fraction. The pellet was suspended in nuclear fraction buffer (20 mM Hepes, 1.5 mM MgCl_2_, 300 mM NaCl, 0.25 mM EDTA, 0.25 mM EGTA, 25% glycerol), incubated for 20 min on ice under agitation, and centrifuged for 20 min at 13,000 g and 4°C. The supernatant was collected as the nuclear-protein enriched fraction. Protein quantification was measured by the Coomassie blue method [33] with bovine serum albumin as the standard. Thirty micrograms of protein in sample buffer were separated by SDS-PAGE on a 10% polyacrylamide gel at a constant voltage of 140 V. Then proteins were transferred onto a nitrocellulose membrane (Thermo-scientific) in a wet system overnight at constant amperage of 20 mA. For blocking of nonspecific binding, 5% BSA in TBS-T was added for 30 min under agitation at room temperature. The membranes were then incubated with primary antibodies for phospho-β-catenin (Ser33/37/Thr41) (Cell Signaling Tech.), HnRNPK (Santa Cruz Biotechnology, Santa Cruz, USA) and GAPDH (Invitrogen, Life Technologies, Carlsbad, USA) overnight at 4°C. Membranes were then washed and probed with the respective secondary antibodies for 1 h under agitation at room temperature. Primary and secondary antibodies were diluted in 5% BSA and TBS-T. Membranes were washed in TBS-T and scanned with Typhoon™ – GE Healthcare. The resulting bands were subjected to densitometric analysis with the ImageJ software. Phospo-β-catenin levels were normalized by comparison to HnRNPK and GAPDH in nuclear and cytosolic fractions, respectively.

### Immunofluorescence Studies

Cells were fixed for 20 min with paraformaldehyde 4% in PBS. Following 45 min of incubation, blocking solution was added containing 2% goat serum combined with 0.1% Triton X100 (Sigma Aldrich Corp., St. Louis, USA) in PBS. In the sequence, cells were incubated for 2 h with anti phospho-β-catenin (Ser33/37/Thr41) (Cell Signaling Tech.) primary antibodies diluted in PBS plus 0.1% Triton X-100 and 2% goat serum. After rinsing with PBS, Alexa fluor 555 rabbit anti-mouse IgG (1∶100) secondary antibody was added for 1 h, followed by a 5-min incubation with DAPI (4′-6-diamino-2-phenylindole). Cells were rinsed with PBS, mounted on slides with D.P.X. (Sigma Aldrich Corp., St. Louis, USA), and examined using an inverted fluorescence microscope (Nikon Eclipse TE 300).

**Figure 1:**
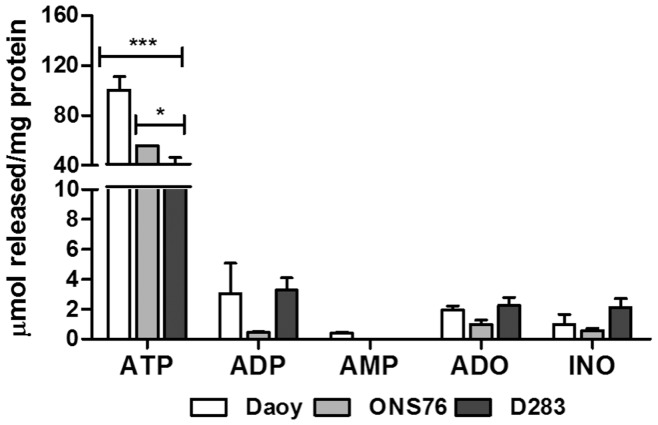
Basal nucleotide secretion by MB cell lines. MB cell lines were incubated for 90 minutes with incubation medium in the absence of substrate, as described in Materials and Methods. Extracellular nucleotide/nucleoside concentrations (ATP, ADP, AMP, ADO and INO) were measured by HPLC and concentrations were expressed as µmol product/mg protein (mean±S.D.). Experiments were performed three times in triplicate and the data obtained were analyzed for statistical relevance using a Two-Way ANOVA test followed by the Bonferroni post hoc test. (***) p<0.001 and (*) p<0.05 compared to control data.

**Figure 2:**
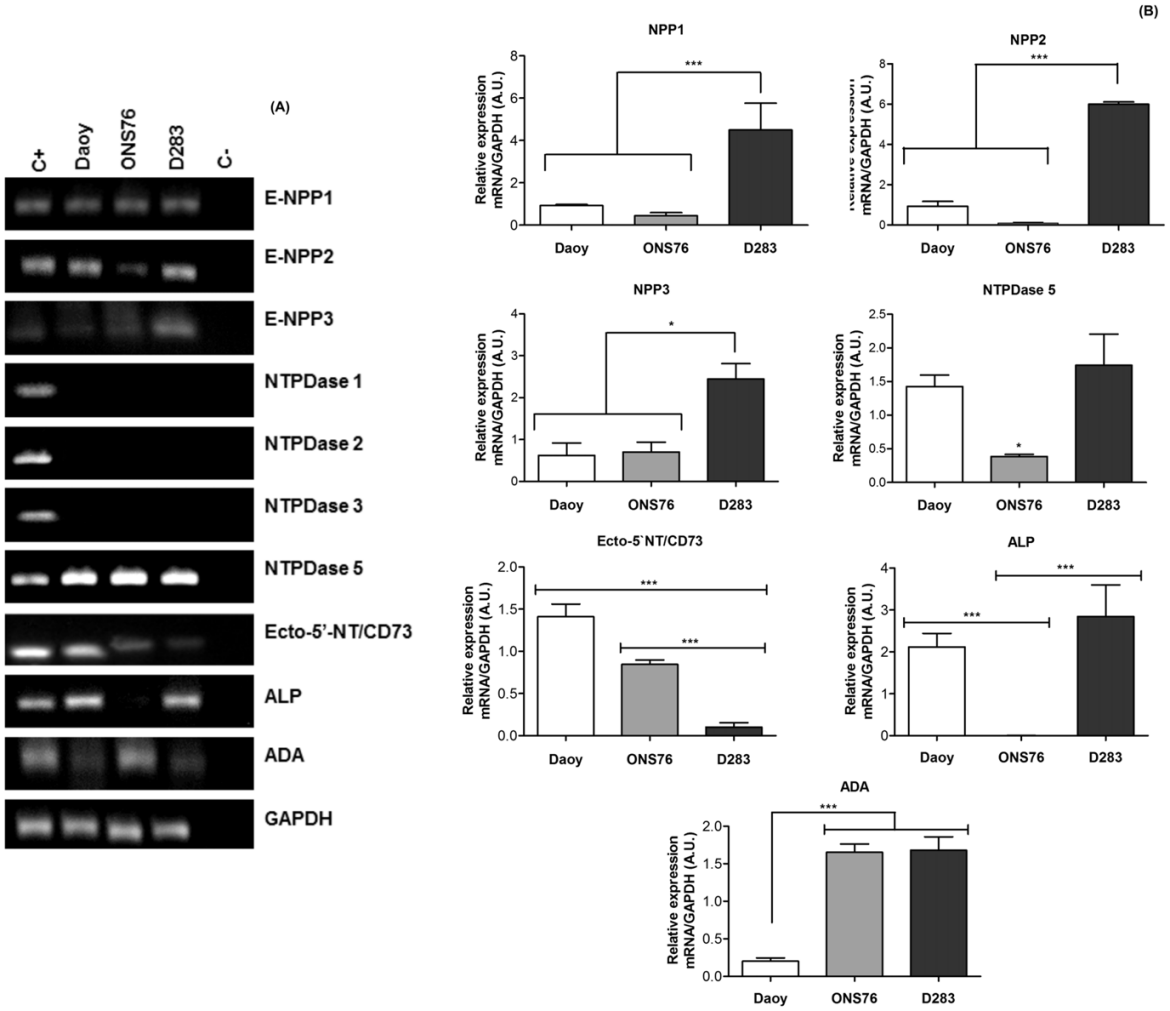
Analysis of ectonucleotidases expression in MB cell lines. Total RNA isolated from cell cultures. RT-PCR and real time-PCR reactions were performed as described in Materials and Methods. (**A**) RT-PCR analysis of E-NPPs, NTPDases, Ecto-5′-NT/CD73, ALP and ADA (**B**) Quantitative analysis of the relative expression of E-NPPs, NTPDases, Ecto-5′-NT/CD73, ALP and ADA in MB cell lines were performed by real time-PCR where GAPDH expression was used as internal control for normalization of expression levels. Data were analyzed by the One-Way ANOVA test followed by the Tukey post hoc test. The experiments were performed three times in triplicate with (* p<0,01) and (*** p<0.001) indicating statistical relevant difference compared to control data.

### Statistical Analysis

Data were expressed as mean ± SD of at least three independent experiments and were subjected to one-way analysis of variance (ANOVA) followed by Tukey post-hoc test or two-way ANOVA followed by Bonferroni test or Tukey post-hoc, when necessary. Differences between mean values were considered significant when *p<0.05*.

## Results

### Adenine Nucleotide/nucleoside Secretion by Daoy, ONS76 and D283 MB Cells

MB cells were cultured and used for determination of adenine nucleotide/nucleoside secretion. All three analyzed cell lines predominantly secrete ATP to the extracellular medium (Figure 1). The Daoy cell line presented a higher secretion (100.04 µMol ±14.99) than the ONS76 and D283 MB cell lines (55.61 µMol ±0.145 and 26.48 µMol ±10.54, respectively). It is important to note that the three MB cell lines secreted ADP and ADO to the extracellular medium, although in much lower concentrations than secreted ATP. Differences in secretion between MB cell lines were not statistically significant. The detected INO probably resulted the deamination of ADO by ADA present in these cells.

**Figure 3:**
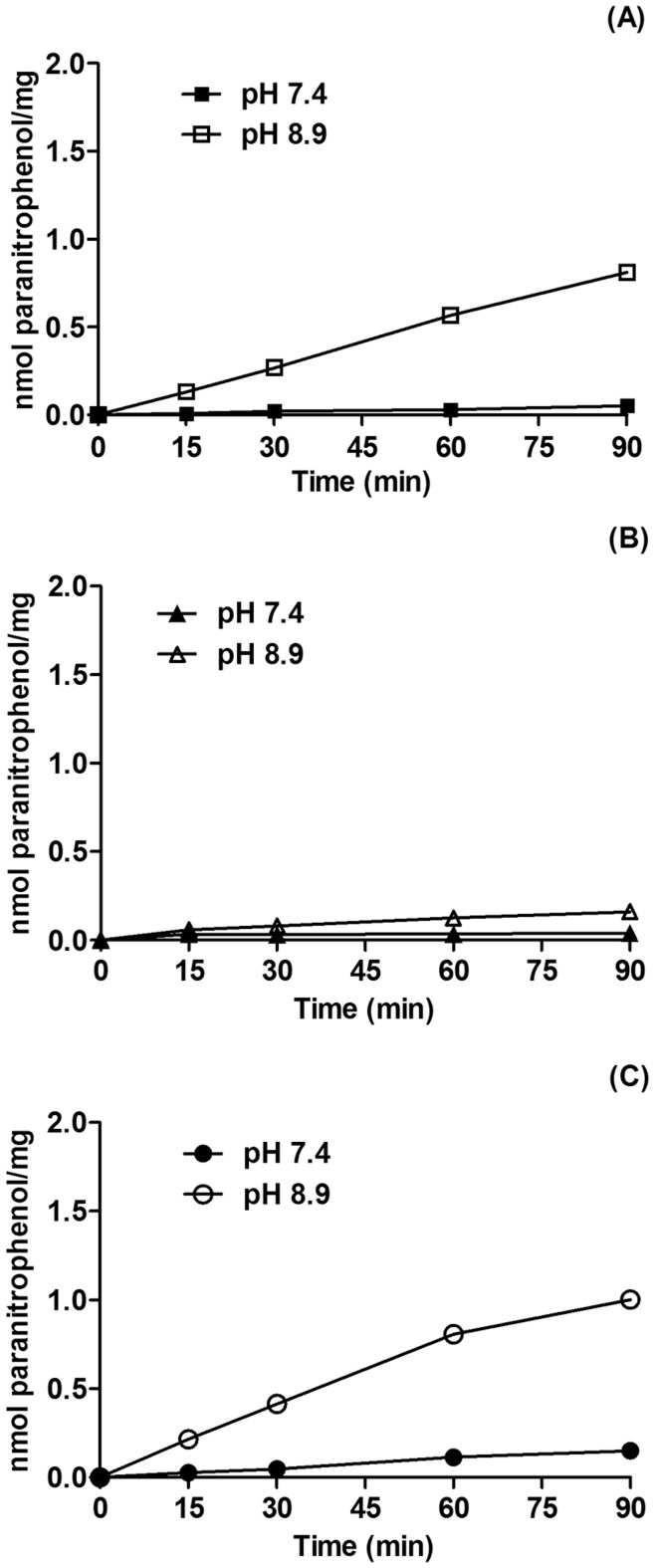
Time kinetics of ecto-nucleotide pyrophosphatase/phosphodiesterase activity by MB cell lines. Following reaching confluence, Daoy (**A**), ONS76 (**B**) and D283 (**C**) MB cell were incubated with 0.5 mM 5′-TMP-p-Nph as described in Materials and Methods. The values represent mean ± SD from six independent experiments performed in triplicate. Specific activities were expressed as nmol ρ-nitrophenol/min/mg of protein.

### Evaluation of Ectonucleotidase mRNA Transcription

ATP transduces a variety of signals into adjacent cells, and these effects are controlled by ectonucleotidase activities [15]. Considering that MB cell lines secrete ATP into the extracellular medium, mRNA transcription of ectonucleotidases in these cell lines was verified by RT-PCR (Figure 2A) and real time-PCR (Figure 2B). We observed that all MB cell lines expressed only NTPDase5 mRNA, but NTPDase1, 2 and 3 gene expression could not be detected (Figure 2A). Real time PCR analysis showed prominent expression of NTPDase5 in D283 cells when compared to other cell lines (Figure 2B). Furthermore, the D283 cell line revealed E-NPP1, 2 and 3 amplicons with expected sizes; real-time PCR confirmed that these expression profiles were significantly higher than those in other MB cell lines. The Daoy and ONS76 MB cell lines expressed all three types of E-NPPs, but in very low proportions, not differing significantly between them.

**Figure 4:**
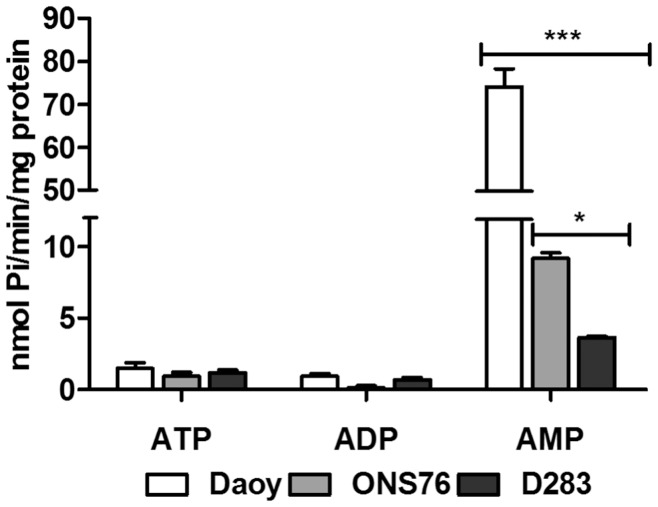
ATP, ADP and AMP hydrolysis by human MB cell lines. Confluent cultures of Daoy, ONS76 and D283 cells were incubated with ATP or ADP or AMP as described in the Materials and Methods section. For D283 cells, a concentration of 2.0 mM and an incubation time of 30 minutes were used for all substrates, whereas for Daoy and ONS76 cell lines, 1.0 mM and 30 minutes of incubation to ATP and ADP, and 2.0 mM and 10 minutes of incubation to AMP were used. Specific activities were expressed as nmol Pi/min/mg of protein. Bars represent mean ± SD of four independent experiments performed in triplicate. Data were compared by Two-Way ANOVA test following by Bonferroni post hoc test. (***) p<0.001 and (*) p<0.05 compared to control data.

Next, we evaluated mRNA transcription of enzymes participating in extracellular ATP metabolism. Ecto-5′NT/CD73 mRNA expression in the primary MB cell lines (Daoy and ONS76) was higher than in the secondary MB cell line (D283). Daoy and D283 cell lines expressed ALP mRNA in similar proportions, and ONS76 and D283 cell lines showed similar ADA expression profiles, both higher than those of the Daoy cell line (Figure 2A and 2B).

**Figure 5:**
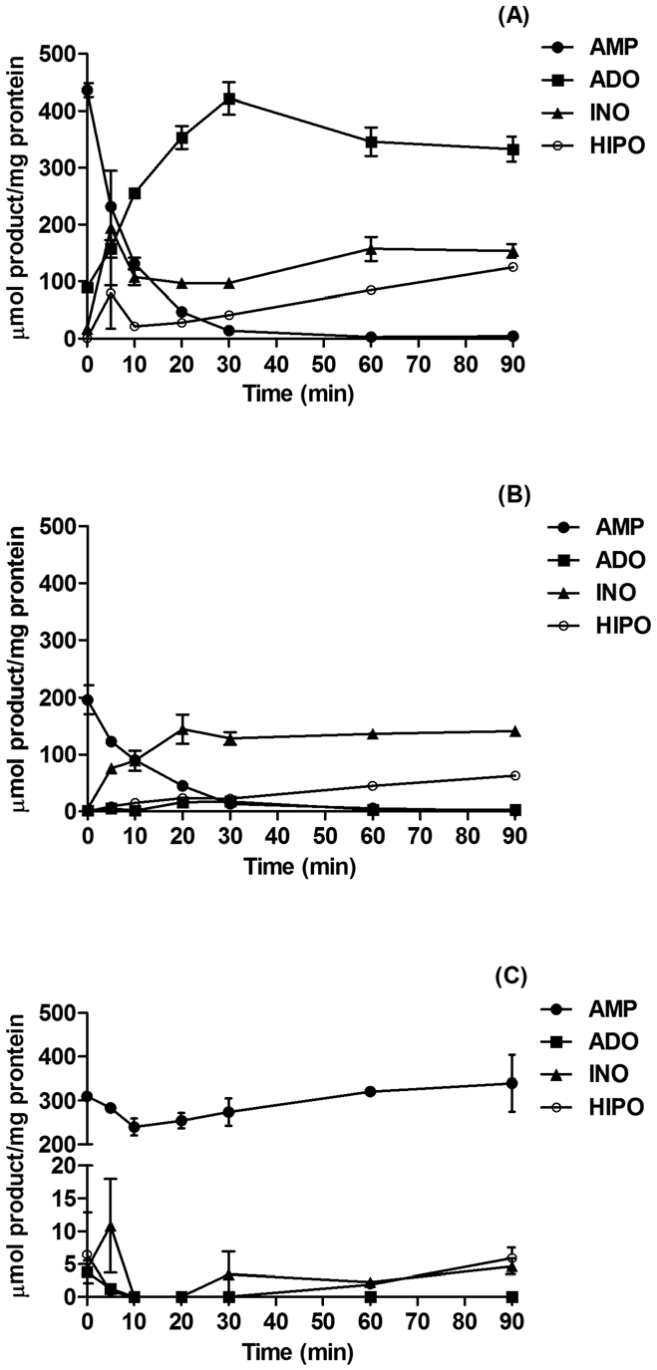
Metabolism of extracellular AMP by MB cell lines. Confluent cultures of Daoy (**A**), ONS76 (**B**) and D283 cells (**C**) were cultivated in 24 well plates until confluence and incubated with 100 µM AMP in 250 µL of incubation medium as described in Materials and Methods. Aliquots of the supernatant were collected at 0, 5, 10, 20, 30, 60 and 90 min, and levels of AMP, ADO, INO and HIPO (hipoxantine) were determined by HPLC. Data were expressed as mean ± SD values from two independent experiments performed in triplicate.

### Ecto-nucleotide Pyrophosphatase/phosphodiesterase (E-NPP) Activity

Considering that E-NPPs can help to keep the ideal nucleotide levels in the extracellular medium [34] and that its expression was detected in the MB cell lines here analyzed, we evaluated the activity of these enzymes as described in Material and Methods. The three cell lines studied showed greater E-NPP activity in alkaline pH, the optimum pH for these enzymes *in vitro* [35]. However, at physiological pH, E-NPP enzymatic activity was extremely low in the MB cell lines, indicating its participation in the extracellular nucleotide metabolism even under these conditions (Figure 3).

**Figure 6:**
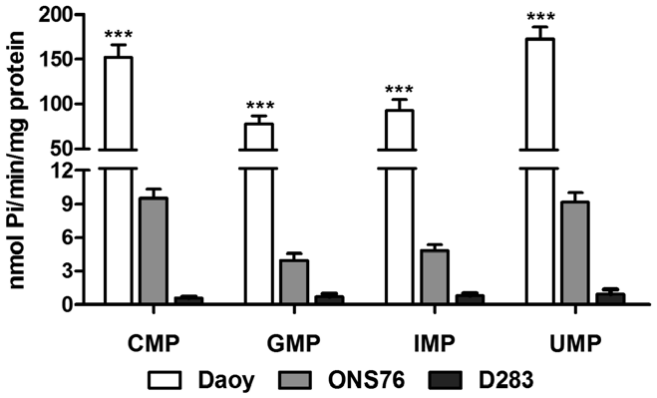
Substrate specificity. Following confluence Daoy, ONS76 and D283 MB cell lines were incubated with different monophosphonucleosides, as described in Materials and Methods section. Bars represent mean ± SD of 6 independent experiments performed in triplicate. Specific activities were expressed as nmol Pi/min/mg of protein. Data were compared by Two-Way ANOVA test following by Bonferroni post hoc test. (***) p<0.001 and was taken to indicate statistical significance.

**Figure 7:**
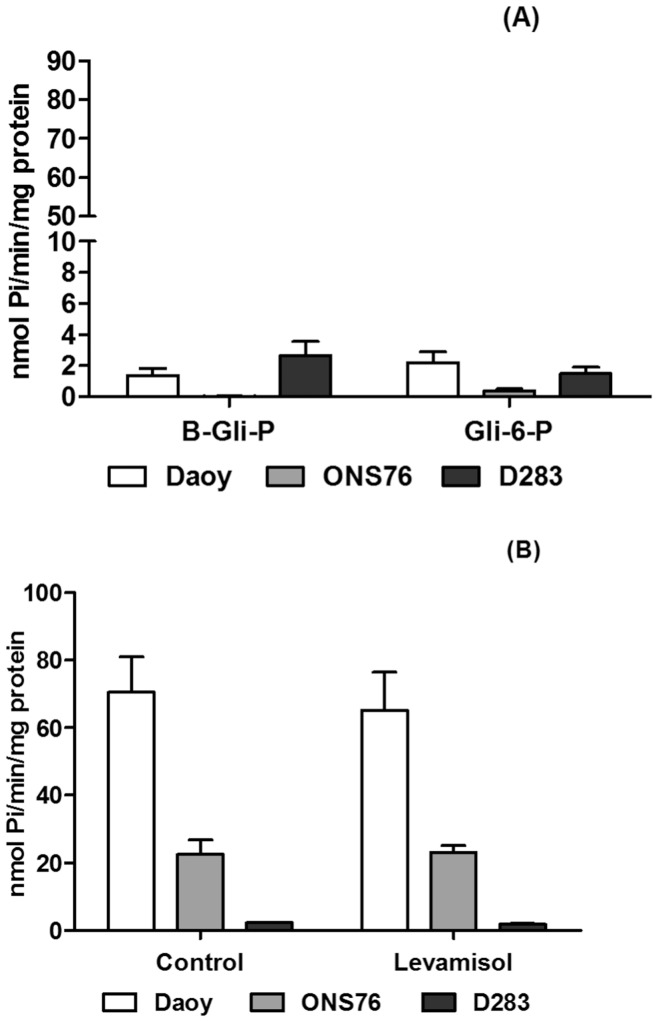
Extracellular hydrolysis of phosphate esters by MB cell lines. After the confluence, the MB cells were incubated and the inorganic phosphate amount was analyzed as described in the Materials and Methods. (**A**) β-glycerophosphate (β-Gli-P), glucose-6-phosphate (Gli-6-P) and inorganic pyrophosphate (PPi), specific substrates to unspecific phosphatases were used to evaluate the enzymatic activity of these enzymes. (**B**) The MB cells were incubated with AMP in the presence of levamisole, a specific alkaline phosphatase inhibitor, to evaluate the influence of the phosphatases activity in AMP hydrolysis. Specific activities were expressed as nmol Pi/min/mg of protein. Data were compared by Two-Way ANOVA test following by Bonferroni post hoc test. All experiments were performed in triplicate.

### ATP, ADP and AMP Hydrolysis

Next, we aimed to analyze E-NTPDase enzymatic activities. For this purpose, differential hydrolysis pattern of ATP, ADP and AMP by Daoy, ONS76 and D283 cell lines were investigated. All MB cell lines presented a low rate of hydrolysis of ATP and ADP (Figure 4). However, when we analyzed AMP hydrolysis, the enzymatic activities of the MB cell lines exhibited a specific and differential profile with Daoy > ONS76> D283. The specific activities were 74.11±8.33, 9.206±0.730 and 3.654±0.157 nmol Pi/min/mg protein, respectively, showing the highest AMPase activities in the Daoy cell line.

**Figure 8:**
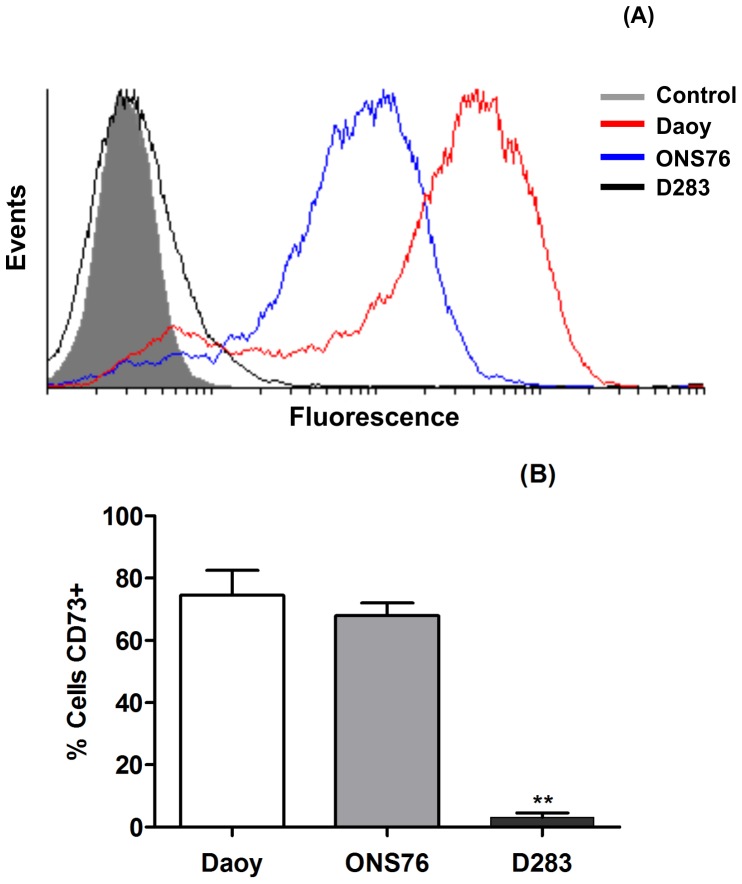
Flow cytometry analysis of ecto-5′-NT/CD73 protein expression in Daoy, ONS76 and D283 MB cell lines. Confluent MB cell lines were trypsinized and prepared as described in Materials and Methods. After incubation with purified mouse anti-human CD73 antibody (1∶10) and labeling with Alexa fluor 555 Rabbit anti-mouse IgG (1∶100), cells were analyzed by flow cytometry. (**A**) Results are shown as the ratio of labeled over non-labeled cells. (**B**) Representative graphic of ecto-5′NT/CD73-positive cells. Statistical relevance was analyzed by the One-Way ANOVA test following by the Tukey post hoc test. The experiments were performed in triplicate. (**) p<0.001 compared to control data.

### Analysis of Extracellular AMP Metabolism by HPLC

AMPase activity of MB cell lines was further studied by HPLC. Figure 5 shows that AMP was efficiently hydrolyzed by Daoy and ONS76 MB cell lines. These cells hydrolyze AMP almost entirely after 30 min of incubation; however, the profiles of hydrolysis were different. While Daoy converted AMP into ADO and accumulated this molecule at the end of the incubation time, ONS76 produced ADO, which probably was converted into INO; this molecule was accumulated as the main product of AMP metabolism. Confirming the results obtained by the colorimetric assay (Fig. 4), the D283 MB cell line did not hydrolyze AMP.

**Figure 9:**
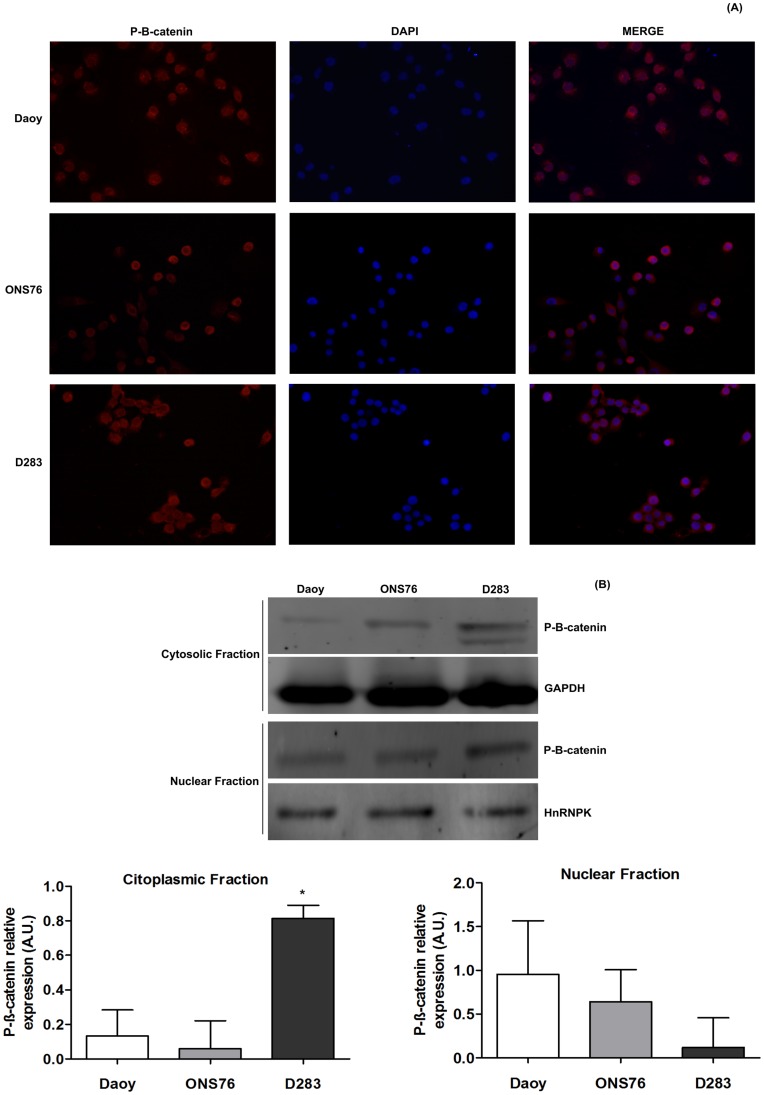
Cytosolic and nuclear P-β-catenin expression in MB cell lines. Phospho-β-catenin expression was determined by (**A**) Immunofluorescence and (**B**) Western blotting assays as described in Materials and Methods. For immunofluorescence, images were captured at objective lens of 20×magnification and the cytosolic and nuclear Phospho-β-catenin immunoreactivity were observed at merge column. For Western blotting, relative Phospho-β-catenin levels of the cytosolic and nuclear fractions obtained by densimetric analysis of protein bands detected in Western blots were compared to GAPDH and HnRNPK expression levels, respectively. Bars represent mean ± SD of 3 independent experiments performed in triplicate. Data were compared by One-Way ANOVA test following by the Tukey post hoc test. (* p<0,01) compared to control data.

### Hydrolysis of Other Monophosphonucleosides

Similar to AMP hydrolysis, the MB cell lines exhibited the same pattern of hydrolysis for other monophosphonucleosides (CMP, GMP, IMP and UMP) (Figure 6), as expected to ecto-5′NT/CD73 [17]. As it was shown before, Daoy and D283 expressed ALP mRNA, another enzyme that can hydrolyze AMP and other phosphate esters. Daoy and ONS76, which presented higher AMPase activity, revealed very low enzymatic activity in the presence of Glu-6P or β-Gly-P, two susbtrates for ALP (Figure 7A). D283 MB cells also poorly hydrolyzed these substrates. In addition, the potential participation of ALP activity in hydrolysis of AMP was evaluated by the presence of levamisole, a specific inhibitor of this enzyme (Figure 7B). AMPase activity in the MB cell lines was unchanged in the presence of the inhibitor, excluding ALP participation in AMP hydrolysis. We observed prominent PPi hydrolysis by all MB cell lines (Figure 7A). Inorganic pyrophosphatase activity was also detected in the three cell lines (Fig. 7A), being in agreement with the presence of an ecto-pyrophosphatase in all MB cell lines analyzed here.

### Analysis of the Ecto-5′NT/CD73 Protein Expression by Flow Cytometry

To confirm the presence of ecto-5′NT/CD73 on the cell surface, we performed flow cytometry analysis as described in the Material and Methods section. Figure 8A confirms the data presented in the mRNA expression analysis, where the cell lines representative of primary MB (Daoy and ONS76) showed higher expression of ecto-5′NT/CD73 than the D283 MB cell line, representative of secondary MB. The results were further quantified and presented the following percentages of ecto-5′NT/CD73 membrane expression in each MB cell line: Daoy: 74.52% ±8.026, ONS76∶67.97±4.087 and D283∶3.115±1.393.

### Phospho-β-catenin MB Cell Expression

β-catenin is a downstream effector of the Wnt canonical pathway and, as described before, it is important for MB tumor prognosis and staging [3], [5]. We used immunofluorescence and Western blotting assays for detecting phospho-β-catenin expression in the MB cell lines used here. Firstly, we observed that Daoy and ONS76 showed mainly nuclear immunoreactivity to phospho-β-catenin, while the D283 MB cell line revealed immunoreactivity for this protein in the cytosol (Figure 9A). These qualitative data were confirmed by Western blotting of cytosolic and nuclear fractions compared to their respective controls, GAPDH and HnRNPK (Figure 9B).

## Discussion

In the present work, we have studied expression and activity of ectonucleotidases in three different medulloblastoma (MB) cell lines (Daoy, ONS76 and D283). ATP is released into the extracellular medium under physiologic or pathologic conditions, stimulating various types of signaling pathways [12]. The analyzed MB cell lines secreted ATP into the extracellular medium in a micromol rate. The obtained data are in line with previous results of our group with the U138MG glioma cell line which also secreted ATP [36]. Moreover, extracellular ATP at 100 µM stimulated glioma cell proliferation [26], while being cytotoxic to rat organotypic hippocampal slice cultures [24].

Once in the extracellular medium, ATP is subject to degradation by ectonucleotidases. The results presented here show that MB cell lines do not express E-NTPDases, with exception of NTPDase5. These data are in accordance with those of malignant gliomas, which present very low ATPase and ADPase activities when compared to their normal counterparts, the astrocytes [23]. The presence of a dominant NTPDase2 in primary astrocyte cultures from hippocampus, cortex and cerebellum [25] may indicate a loss in E-NTPDase expression/activity during the malignant transformation of brain tumors. Similarly, NTPDase5, an intracellularly expressed enzyme considered to act as a proto-oncogene, is not present in normal astrocytes. Consequently, its expression rate may correlate with the degree of malignancy [11], [37].

E-NPPs also hydrolyze di- and tri-phosphate nucleotides in the extracellular medium. Various tissue types express these enzymes, which are associated with a variety of pathophysiological and biochemical functions [35], [38]. Here, we demonstrate that Daoy and ONS76 reveal low expression and activity of all analyzed E-NPPs when compared to the D283 cell line. D283 cells expressed all E-NPPs in the following order: NPP2> NPP1> NPP3. NPP3 expression was observed in immature astrocytes of pre-natal and neonatal rats, collaborating with other proteins for glial cell tumor transformation following treatment with N, N’-ethylnitrosourea [35]. Therefore, our hypothesis is that expression this enzyme is correlated with an undifferentiated cell stage. NPP1, a suggest marker for glioma malignancy, was present in the rat glioma C6 cell line, where together to NPP3, was responsible for hydrolyzing low ATP concentrations in the C6 glioma cell line (1–10 µM) [39]. In the study we show that E-NPP activity was also present at low levels as judged by hydrolysis rates of its specific artificial substrate, 5′-TMP-p-Nph. These results together with the absence of E-NTPDase expression, is in line with low hydrolysis rates of ATP and ADP nucleotides, and this may be attributed to E-NPP activity.

NPP2 exerts numerous functions including promotion of angiogenesis, cell proliferation and differentiation. NPP2 was the most prominent E-NPP enzyme expressed by the D283 cell line, which was derived from a metastatic tumor. This observation agrees with previously published reports suggesting functions of NPP2 as stimulator of tumor motility in melanoma cells and in breast cancer. Furthermore, expression of this enzyme was up-regulated in lung cancer and related to the degree of growth and invasiveness [35], [40]. This data, associated with ATP secretion presented by MB cell lines is in accordance with the current literature, suggesting that ATP is accumulated in the extracellular space of MB cells.

An important finding of our present work is that primary MB tumor cells, such as Daoy and ONS76 lines express ecto-5′NT/CD73 while the D283 cell line, representative of a metastatic tumor, showed very low expression of this enzyme. These results are intriguing considering that increased ecto-5′NT/CD73 expression/activity is the most evident alteration in the ectonucleotidase pathway related to malignancy grades of different tumor types, including gliomas [8]. In addition, we showed that the three MB cell lines express the ecto-adenosine deaminase (ADA) in an inverse order compared to the expression profile of ecto-5′NT/CD73, confirmed by differences in adenosine and inosine production, as demonstrated in Figure 5.

A possible explanation for this inversion of expression/activity patterns of ecto-5′NT/CD73 and ADA in MB cells may be provided by the Wnt/β-catenin pathway. Wnt/β-catenin induced signal transduction regulates the expression of several genes relevant for cancer, including MB [41]. Briefly, β-catenin cytoplasmic levels may be controlled by the Wnt pathway, since it can destabilize a multimeric protein complex in the cytoplasm, leading to augmented levels of free β-catenin, which undergoes nuclear translocation. In the pathway inactive state, GSK-3β and APC, both present in the cytosolic protein complex, phosphorylate cytosolic β-catenin, which then becomes ubiquitinated for subsequent cytoplasmic degradation [7], [42]. Mutations of APC were detected in a wide variety of human cancers, including MB. Deficient β-catenin phosphorylation by APC impairs its cytoplasmic degradation and consequently favors phospho-β-catenin translocation to nucleus [42]. In addition, cytoplasmic immunoreactivity for β-catenin in tumor tissue samples of patients with MB was associated with a poor prognosis, while nuclear immunoreactivity for β-catenin was associated with a better prognosis, with patients demonstrating a greater life expectancy [7].

The expression of ecto-5′NT/CD73 and ADA is inversely regulated by interactions between β-catenin and TCF/LEF, resulting in a nuclear protein complex which interacts with the promoter region of these genes. In agreement, transfection of β-catenin and TCF-1 genes in Rat-1 cells augmented ecto-5′NT/CD73 and a decrease ADA activity and expression [43]. Our results showed that β-catenin nuclear immunoreactivity was higher in Daoy and ONS76 cell lines than in D283 cells. Considering that the metastatic phenotype is associated with poor patient prognosis [41], we suggest that the absence of ecto-5′NT/CD73 and the presence of ADA in the D283 cell line, a metastatic MB phenotype, may be related with its poor prognosis. Further studies are needed to fully elucidate the participation of these enzymes in MB progression.
