# Correction: Characterization of Ectonucleotidases in Human Medulloblastoma Cell Lines: ecto-5′NT/CD73 in Metastasis as Potential Prognostic Factor

**DOI:** 10.1371/annotation/0e219081-9218-480c-aa54-1142a68aed14

**Published:** 2012-11-06

**Authors:** Angélica Regina Cappellari, Liliana Rockenbach, Fabrícia Dietrich, Vanessa Clarimundo, Talita Glaser, Elizandra Braganhol, Ana Lúcia Abujamra, Rafael Roesler, Henning Ulrich, Ana Maria liveira Battastini

The final author's name was spelled incorrectly. The correct name is: Ana Maria Oliveira Battastini.

